# Factors associated with self-reported falls, balance or walking difficulty in older survivors of breast, colorectal, lung, or prostate cancer: Results from Surveillance, Epidemiology, and End Results–Medicare Health Outcomes Survey linkage

**DOI:** 10.1371/journal.pone.0208573

**Published:** 2018-12-19

**Authors:** Min H. Huang, Jennifer Blackwood, Monica Godoshian, Lucinda Pfalzer

**Affiliations:** 1 Physical Therapy Department, College of Health Sciences, University of Michigan–Flint, Flint, MI, United States of America; 2 Department of Physical Medicine and Rehabilitation, Michigan Medicine, University of Michigan, Ann Arbor, MI, United States of America; West Chester University of Pennsylvania, UNITED STATES

## Abstract

**Background:**

Cancer and its treatment affect body systems that are important in preventing falls and controlling balance/walking. This study examined factors associated with self-reported falls and balance/walking difficulty in the past 12 months in older survivors of four major cancers.

**Methods:**

This was a cross-sectional study analyzing population-based data from Surveillance, Epidemiology, and End Results–Medicare Health Outcomes Survey (SEER-MHOS). Data from cohorts 9 to 14 (January 2006 to December 2013) were extracted. Inclusion criteria were: age ≥65 years at cancer diagnosis, first MHOS completed during years 1–5 post-cancer diagnosis, first primary breast (n = 2725), colorectal (n = 1646), lung (n = 752), and prostate (n = 4245) cancer, and availability of cancer staging information. Primary outcomes were self-reported falls and balance/walking difficulty in the past 12 months. Multivariable logistic regression was constructed for each cancer type to examine independent factors associated with falls and balance/walking difficulty.

**Results:**

In all cancer types, advancing age at cancer diagnosis and dependence in activities of daily living were significant independent factors associated with increased odds of reporting falls and balance/walking difficulty in the past 12 months. Additionally, depression was independently associated with falls and sensory impairment in feet was independently linked to balance/walking difficulty in all cancer types. Other independent factors of falls and balance/walking difficulty varied across cancer types. In breast cancer only, localized or regional cancer stage was significantly associated with increased odds of reporting falls and balance/walking difficulty, whereas treatment with radiation decreased the odds of falling. No association between falls and balance/walking difficulty with time since cancer diagnosis, cancer stage, or cancer treatment was found in colorectal, lung, and prostate cancer.

**Conclusion:**

There exists some heterogeneity in factors associated with self-reported falls and balance/walking difficulty between different cancer types. Future research is necessary to ascertain factors predictive of falls and balance/walking difficulty in older cancer survivors, particularly factors related to cancer diagnosis and treatment.

## Introduction

Falls and balance/walking difficulty are significant concerns in older adults. Nearly one in four adults aged ≥65 years fall annually [1]. Among those who fall, 20%-30% sustain serious injuries [2]. While falls are multifactorial, balance and gait disorders are major risk factors [3]. Falls and balance/walking difficulty precipitate institutionalization, functional declines, activity restrictions, fears of falling, and recurrent falls [2,4–7].

A cancer survivor is a person from the time of cancer diagnosis until death [8]. Approximately 50% of new cancers each year are diagnosed in people aged ≥65 years [9]. Research suggests that fall rates are higher in older cancer survivors than non-cancer individuals (33% vs. 30% in a community-dwelling sample [10] and 26% vs. 22% among Medicare beneficiaries [11]). In a study of cancer survivors in outpatient setting, balance/walking difficulty (19.4%-23.9%) was identified as the leading functional problem [12]. Older women post-cancer diagnosis <5 years were more likely to report “unable to walk a half mile” than non-cancer controls (ORs = 1.34–1.92) [13]. In older cancer survivors, faster walking speed predicted lower mortality [14]. Walking likely reflects function of physiologic systems that may be affected by cancer.

In older cancer survivors, falls and balance/walking difficulty are linked to poor quality of life [15,16]. Common risk factors for falls in older adults, including age, gender, polypharmacy, and opioid use are not predictive of falls in older cancer survivors [17,18]. A history of falls, impaired cognition or vision, psychotropic drugs, comorbidity, and balance/walking disorders are associated with falls in cancer survivors [17,19–23]. Balance/walking difficulty in survivors were primary examined in the context related to cancer treatment. Breast cancer survivors had reduced balance after Taxane chemotherapy [24]. Prostate cancer survivors receiving androgen deprivation therapy (ADT) had greater declines in balance over 3 years than survivors without ADT or non-cancer controls [25]. Research about factors linked to balance/walking difficulty in cancer survivors is sparse.

Sequelae associated with cancer and cancer treatments vary widely across cancer types [26–28]. As such, investigating factors linked to falls and balance/walking difficulty in each cancer is necessary. Using population-based data, this study aimed to identify factors linked to self-reported falls and balance/walking difficulty in the past 12 months in older survivors of breast, colorectal, lung, and prostate cancer.

## Methods

### Design and data

This observational, cross-sectional study was approved by Institute Review Board of University of Michigan-Flint. SEER includes cancer-related information, such as cancer diagnosis, cancer stage, time of diagnosis, histology, and cancer treatment, except for chemotherapy and hormonal therapy [29]. MHOS collects demographics and patient-reported outcomes in health, function, symptoms, and quality of life [29,30]. Centers for Medicare & Medicaid Services administers MHOS to randomly selected beneficiaries of Medicare Advantage Organizations each year, and participants are resurveyed 2 years later [29,30].

### Participants

**Fig 1:**
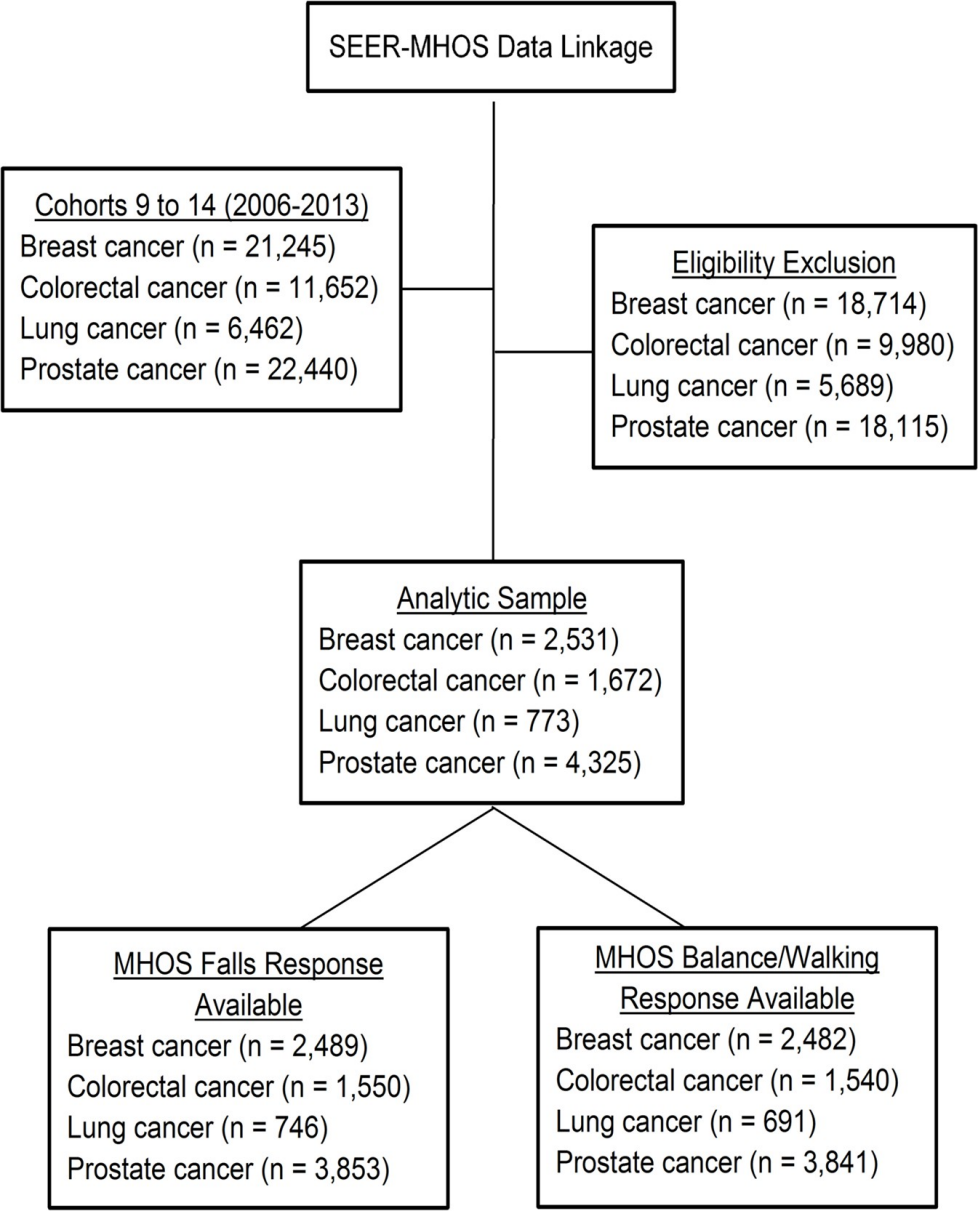
Flowchart of study sample.

We extracted data from cohorts 9–14 (January 2006-December 2014) from SEER-MHOS linkage. Inclusion criteria were: age at cancer diagnosis ≥65 years, first primary breast, colorectal, lung, or prostate cancer, first MHOS completed from each participant during years 1–5 post-cancer diagnosis, and availability of cancer staging information. Applying these criteria resulted in 9,540 survivors (breast = 2,725, colorectal = 1,646, lung = 752, and prostate cancer = 4,245). These four cancer diagnoses were chosen because they are the most prevalent cancer types in adults aged 65 years and over [31]. Additionally, previous research has indicated that the prevalence of falls and balance/walking difficulty increased from pre- to post-cancer diagnosis in older breast, lung, and prostate cancer survivors [32]. Fig 1 shows sample flowchart.

### Primary outcomes

Falls and balance/walking difficulty were determined by two MHOS questions: (1) “A fall is when your body goes to the ground without being pushed. Did you fall in the past 12 months?” (2) “In the past 12 months, have you had a problem with balance or walking?” Responses were “no” (coded as 1) or “yes” (coded as 2).

### Variables

Table 1 provides detailed descriptions of variables. Demographics included gender, race, and marital status. Health-related variables included number of comorbidities (arthritis, osteoporosis, angina, congestive heart failure, myocardial infarction, stroke, hypertension, emphysema/asthma/chronic obstructive pulmonary disease, diabetes, low back pain, and obesity), limitation in moderate activities, number of activities of daily living (ADL) dependence, fatigue, depression, urinary incontinence, visual, hearing, or sensory impairments, and pain interfering with work. Cancer-related variables included age at cancer diagnosis, time since cancer diagnosis, cancer type, stage, surgery, and radiation. The time since cancer diagnosis was from the time of cancer diagnosis to the time when a participant completed the MHOS.

**Table 1:** List of variables, detailed definition, and coding.

Variable	Definition	Coding
Gender	Survey reported gender.	1 = Men 2 = Women
Race	Race from enrollment data base maintained by Center for Medicare and Medicaid Services.	1 = White 2 = Black or other race
Marital status	Survey reported marital status.	1 = Married 2 = Divorced, separated, widowed, or never married
Limitation in moderate physical activity	Health limiting one in moderate activities during a typical day, such as moving a table, pushing a vacuum cleaner, bowling, or playing golf.	1 = Not limited or limited a little 2 = Yes limited a lot
Activities of daily living (ADL) dependence	Difficulty doing six ADL without special equipment or help from another person (bathing, dressing, eating, getting in or out of chairs, walking, and using toilet).	1 = Independent (no difficulty) 2 = Dependence in one or more ADL (have difficulty or unable to perform the activity)
Fatigue	Fatigue during the past 4 weeks.	1 = No fatigue 2 = Yes fatigue
Urinary incontinence	Accidentally leaked urine in the last 6 months.	1 = No 2 = Yes
Vision problem	Able to see well enough to read newspaper print (with glasses or contacts if that’s how one sees best).	1 = Yes 2 = No
Hearing problem	Able to hear most of the things people say (with a hearing aid if that’s how one hears best).	1 = Yes 2 = No
Sensory impairment in feet	Numbness or loss of feeling in feet, tingling or burning sensation in feet especially at night, decreased ability to feel hot or cold with feet during the past 4 weeks.	1 = Not impaired 2 = Yes impaired
Pain interfering with work	Pain in the past 4 week interferes with normal work (including both work outside the home and housework)	1 = Not interfering with work 2 = Yes interfering with work
Cancer stage	Surveillance, Epidemiology, and End Results (SEER) summary stage 2000, derived from Collaborative Stage (CS)	1 = In situ 2 = Localized 3 = Regional 4 = Distant
Radiation	Radiation therapy performed as part of the first course of cancer treatment.	1 = No radiation 2 = Yes, with radiation
Surgery	Surgery of primary site that removes and/or destroys tissue of the primary site performed as part of the initial work-up or first course of therapy.	1 = No surgery 2 = Yes, with surgery

### Statistical analysis

Statistical analyses were performed using IBM-SPSS 24 (IBM Corp., Armonk, NY). Analyses were conducted relating to each primary outcome separately for each cancer. Descriptive statistics were calculated for sample characteristics. Univariable logistic regression was performed to examine associations of each factor with primary outcomes. Subsequently, all demographic, health- and cancer-related variables were entered into multivariable logistic regression models to identify independent factors linked to each of the primary outcomes. Multicollinearity was determined by variance inflation factor ≥4 [33]. Model fit was assessed by Hosmer and Lemeshow test [34]. Two-sided significance level was *p*<0.05.

## Results

### Sample characteristics

Average age at MHOS administration was 76.8 (6.3) years for breast, 78.5 (6.7) years for colorectal, 77.1 (5.8) years for lung, and 76.0 (5.6) years for prostate cancer. Average time post-diagnosis was 35.0 (13.7) months for breast, 35.3 (13.8) months for colorectal, 31.2 (13.5) months for lung, and 35.9 (13.8) months for prostate cancer. Percentages of survivors reporting falls in the past 12 months were 25.6% in breast, 23.3% in colorectal, 25.3% in lung, and 19.7% in prostate cancer. Balance/walking difficulty in the past 12 months was reported in 38.4% of breast, 35.3% of colorectal, 45.4% of lung, and 27.5% of prostate cancer survivors.

### Univariable analyses

As shown in Tables 2–5, in breast, colorectal, and prostate cancer, being white and unmarried were associated with increased odds of falls. In colorectal cancer, women were more likely to report falls than men. In all cancer types, all health-related factors were associated with increased falls. Except for lung cancer, advancing age at cancer diagnosis was associated with increased falls. In breath cancer, local or regional cancer stage was associated with increased risk of falls, whereas radiotherapy was associated with decreased falls. On the other hand, surgery in prostate cancer were associated with decreased falls.

**Table 2:** Crude and adjusted odds ratios for self-reported falls and balance/walking difficulty in the past 12 months by demographic, health- and cancer-related factors in breast cancer.

Variables	Falls				Balance or Walking Difficulty			
	No (n = 1852)	Yes (n = 637)	Crude OR (95% CI)	Adjusted OR (95% CI)	No (n = 1529)	Yes (n = 953)	Crude OR (95% CI)	Adjusted OR (95% CI)
Race								
White	1390	510	1.0	1.0	1179	721	1.0	1.0
Other	458	125	**0.74 (0.60–0.93)**^**b**^	**0.65 (0.48–0.87)**^**b**^	347	229	1.08 (0.89–1.31)	0.91 (0.66–1.25)
Marital status								
Married	773	234	1.0	1.0	684	318	1.0	1.0
Not married	1067	391	**1.21 (1.00–1.46)**^**a**^	1.04 (0.82–1.32)	833	623	**1.61 (1.36–1.90)**^**c**^	0.99 (0.76–1.56)
Comorbidity	2.8 (1.7)	3.6 (1.9)	**1.26 (1.19–1.33)**^**c**^	**1.09 (1.02–1.17)**^**a**^	2.5 (1.6)	3.9 (1.8)	**1.61 (1.52–1.71)**^**c**^	**1.19 (1.09–1.29)**^**c**^
Moderate physical activity								
Not limited	1463	383	1.0	1.0	1329	514	1.0	1.0
Limited a lot	352	245	**2.66 (2.09–3.47)**^**c**^	1.11 (0.82–1.51)	169	425	**6.50 (5.30–7.98)**^**c**^	1.10 (0.78–1.56)
ADL dependency								
None	1179	234	1.0	1.0	1200	214	1.0	1.0
1–2 ADL	431	180	**2.10 (1.68–2.63)**^**c**^	1.34 (0.98–1.81)	235	372	**8.88 (7.13–11.04)**^**c**^	**4.92 (3.62-.6.69)**^**c**^
3+ ADL	170	193	**5.72 (4.46–7.34)**^**c**^	**1.97 (1.32–2.93)**^**b**^	46	316	**38.52 (27.37–54.22)**^**c**^	**12.55 (7.65–20.60)**^**c**^
Fatigue								
No	1449	368	1.0	1.0	1307	504	1.0	1.0
Yes	361	251	**2.74 (2.25–3.34)**^**c**^	1.11 (0.83–1.49)	186	425	**5.93 (4.85–7.24)**^**c**^	**1.69 (1.22–2.34)**^**b**^
Depression								
No	1248	283	1.0	1.0	1104	427	1.0	1.0
Yes	398	280	**3.10 (2.54–3.80)**^**c**^	**2.07 (1.61–2.66)**^**c**^	256	418	**4.22 (3.49–5.11)**^**c**^	**2.20 (1.65–2.93)**^**c**^
Urinary incontinence								
No	1029	228	1.0	1.0	908	345	1.0	1.0
Yes	796	399	**2.26 (1.88–2.73)**^**c**^	**1.63 (1.29–2.05)**^**c**^	599	593	**2.61 (2.20–3.08)**^**c**^	**1.64 (1.26–2.13)**^**c**^
Vision problem								
No	1746	556	1.0	1.0	1463	831	1.0	1.0
Yes	90	67	**2.34 (1.68–3.25)**^**c**^	1.03 (0.78–1.36)	48	108	**3.96 (2.79–5.62)**^**c**^	**1.98 (1.09–3.57)**^**a**^
Hearing problem								
No	1599	503	1.0	1.0	1343	750	1.0	1.0
Yes	185	114	**1.96 (1.52–2.53)**^**c**^	1.09 (0.77–1.53)	130	169	**2.33 (1.82–2.98)**^**c**^	1.05 (0.70–1.58)
Sensory impairment								
No	1457	429	1.0	1.0	1283	598	1.0	1.0
Yes	202	138	**2.32 (1.82–2.96)**^**c**^	1.15 (0.84–1.57)	90	248	**5.91 (4.56–7.67)**^**c**^	**1.86 (1.09–1.29)**^**b**^
Pain interfering with work								
No	1091	242	1.0	1.0	1052	284	1.0	1.0
Moderate or Severe	732	386	**2.38 (1.97–2.86)**^**c**^	1.03 (0.78–1.36)	455	653	**5.32 (4.45–6.35)**^**c**^	1.08 (0.80–1.45)
Age at diagnosis, y	72.8 (5.9)	74.7 (6.8)	**1.05 (1.03–1.06)**^**c**^	**1.03 (1.01–1.05)**^**b**^	72.5 (5.7)	74.7 (6.7)	**1.06 (1.05–1.08)**^**c**^	**1.02 (1.00–1.05)**^**a**^
Survival time since cancer diagnosis, m	34.7 (13.7)	35.8 (14.0)	1.01 (0.99–1.01)	1.01 (1.00–1.02)	34.8 (13.8)	35.6 (13.6)	1.00 (1.00–1.01)	1.00 (0.99–1.01)
Cancer stage								
In situ	342	85	1.0	1.0	280	144	1.0	1.0
Localized	1096	399	**1.47 (1.13–1.91)**^**b**^	**1.88 (1.29–2.70)**^**b**^	928	563	1.18 (0.94–1.48)	1.42 (0.99–2.04)
Regional	381	144	**1.52 (1.12–2.06)**^**b**^	**1.74 (1.14–2.64)**^**a**^	300	224	**1.45 (1.11–1.89)**^**b**^	**1.58 (1.04–2.40)**^**a**^
Distant	33	9	1.10 (0.51–2.38)	1.15 (0.39–3.34)	21	22	**2.04 (1.08–3.83)**^**a**^	1.20 (0.42–3.44)
Surgery								
No	44	21	1.0	1.0	28	38	1.0	1.0
Yes	1804	616	0.72 (0.42–1.21)	0.89 (0.39–2.06)	1498	914	**0.45 (0.27–0.74)**^**b**^	0.55 (0.21–1.43)
Radiation								
No	820	344	1.0	1.0	640	520	1.0	1.0
Yes	994	287	**0.69 (0.57–0.83)**^**c**^	**0.75 (0.59–0.96)**^**a**^	855	423	**0.61 (0.52–0.72)**^**c**^	0.80 (0.61–1.04)

Abbreviation: ADL, activities of daily living.

Values show are number, mean (SD), or ORs (95%CI).

Statistically significant ORs are marked in bold text with numerical superscripts indicating the level of significance.

^a^ *p*-value <0.05,

^b^ *p*-value <0.05, and

^c^ *p*-value <0.05.

**Table 3:** Crude and adjusted odds ratios for self-reported falls and balance/walking difficulty in the past 12 months by demographic, health- and cancer-related factors in colorectal cancer.

Variables	Falls				Balance or Walking Difficulty			
	No (n = 1189)	Yes (n = 361)	Crude OR (95% CI)	No (n = 996)	Yes (n = 544)	No (n = 996)	Crude OR (95% CI)	Adjusted OR (95% CI)
Gender								
Men	576	148	1.0	1.0	495	224	1.0	1.0
Women	604	204	**1.31 (1.03–1.67)**^**a**^	1.23 (0.88–1.73)	490	313	**1.41 (1.14–1.75)**^**b**^	1.09 (0.77–1.56)
Race								
White	576	148	1.0	1.0	753	435	1.0	1.0
Other	604	204	**0.73 (0.54–0.99)**^**a**^	0.69 (0.46–1.02)	233	106	0.79 (0.61–1.02)	**0.57 (0.38–0.87)**^**b**^
Marital status								
Married	612	159	1.0	1.0	535	227	1.0	1.0
Not married	557	193	**1.33 (1.05–1.69)**^**a**^	0.98 (0.70–1.38)	443	306	**1.63 (1.32–2.01)**^**c**^	1.13 (0.79–1.62)
Comorbidity	2.5 (1.8)	3.4 (2.0)	**1.25 (1.17–1.34)**^**c**^	**1.13 (1.03–1.23)**^**b**^	2.2 (1.6)	3.7 (1.9)	**1.54 (1.43–1.66)**^**c**^	**1.23 (1.12–1.36)**^**c**^
Moderate physical activity								
Not limited	926	208	1.0	1.0	850	273	1.0	1.0
Limited a lot	245	148	**2.67 (1.87–3.80)**^**c**^	0.92 (0.61–1.38)	130	265	**6.35 (4.94–8.15)**^**c**^	**1.58 (1.05–2.38)**^**a**^
ADL dependency								
None	791	128	1.0	1.0	787	130	1.0	1.0
1–2 ADL	246	104	**2.61 (1.94–3.51)**^**c**^	**1.86 (1.26–2.76)**^**b**^	145	201	**8.39 (6.32–11.14)**^**c**^	**4.63 (3.18–6.76)**^**c**^
3+ ADL	120	106	**5.46 (3.96–7.53)**^**c**^	**3.37 (2.02–5.62)**^**c**^	43	182	**25.62 (17.51–37.49)**^**c**^	**6.39 (3.73–10.92)**^**c**^
Fatigue								
No	916	199	1.0	1.0	833	272	1.0	1.0
Yes	245	153	**2.88 (2.23–3.70)**^**c**^	1.35 (0.92–1.98)	141	257	**5.58 (4.36–7.15)**^**c**^	1.34 (0.90–1.99)
Depression								
No	810	192	1.0	1.0	732	261	1.0	1.0
Yes	264	138	**2.97 (2.46–3.60**)^**c**^	**1.63 (1.16–2.28)**^**b**^	180	220	**3.43 (2.69–4.37)**^**c**^	**1.52 (1.06–2.19)**^**a**^
Urinary incontinence								
No	761	178	1.0	1.0	687	247	1.0	1.0
Yes	397	174	**1.87 (1.47–2.39)**^**c**^	1.06 (0.77–1.46)	290	275	**2.64 (2.12–3.29)**^**c**^	1.10 (0.79–1.54)
Vision problem								
No	1082	312	1.0	1.0	926	458	1.0	1.0
Yes	89	45	**1.75 (1.20–2.56)**^**b**^	0.99 (0.59–1.65)	59	76	**2.60 (1.82–3.73)**^**c**^	1.55 (0.88–2.71)
Hearing problem								
No	961	275	1.0	1.0	839	385	1.0	1.0
Yes	174	81	**1.63 (1.21–2.19)**^**b**^	0.86 (0.57–1.30)	125	131	**2.28 (1.74–3.00)**^**c**^	0.96 (0.62–1.48)
Sensory impairment								
No	941	232	1.0	1.0	827	341	1.0	1.0
Yes	146	101	**2.81 (2.10–3.76)**^**c**^	**1.73 (1.18–2.52)**^**b**^	87	155	**4.32 (3.23–5.78)**^**c**^	**2.64 (1.73–4.04)**^**c**^
Pain interfering with work								
No	752	156	1.0	1.0	723	178	1.0	1.0
Moderate or Severe	425	198	**2.25 (1.76–2.86)**^**c**^	0.89 (0.61–1.30)	264	356	**5.48 (4.36–6.88)**^**c**^	1.39 (0.79–1.54)
Age at diagnosis, y	74.4 (6.6)	76.8 (6.9)	**1.05 (1.04–1.07)**^**c**^	**1.04 (1.01–1.06)**^**b**^	73.9 (6.4)	77.1 (6.8)	**1.08 (1.06–1.09)**^**c**^	**1.04 (1.02–1.07)**^**b**^
Survival time since cancer diagnosis, m	35.2 (13.6)	35.4 (14.2)	1.00 (0.99–1.01)	1.00 (0.99–1.01)	35.0 (13.8)	35.5 (13.8)	1.00 (1.00–1.01)	1.01 (0.99–1.02)
Cancer stage								
In situ	79	26	1.0	1.0	71	43	1.0	1.0
Localized	649	172	0.87 (0.55–1.37)	0.76 (0.41–1.38)	566	292	0.85 (0.57–1.28)	1.08 (0.55–2.12)
Regional	448	160	1.15 (0.72–1.84)	1.18 (0.64–2.20)	380	220	0.96 (0.63–1.45)	1.36 (0.68–2.71)
Distant	54	10	0.60 (0.27–1.33)	0.39 (0.14–1.11)	39	25	1.06 (0.56–1.99)	1.24 (0.44–3.44)
Surgery								
No	27	11	1.0	1.0	24	13	1.0	1.0
Yes	1160	349	0.74 (0.36–1.50)	1.06 (0.47–3.05)	969	531	1.01 (0.51–2.00)	1.57 (0.49–5.03)
Radiation								
No	1067	323	1.0	1.0	891	493	1.0	1.0
Yes	114	34	0.99 (0.66–1.47)	1.08 (0.65–1.82)	100	44	0.80 (0.55–1.15)	0.70 (0.40–1.23)

Abbreviation: ADL, activities of daily living.

Values show are number, mean (SD), or ORs (95%CI).

Statistically significant ORs are marked in bold text with numerical superscripts indicating the level of significance.

^a^ *p*-value <0.05,

^b^ *p*-value <0.05, and

^c^ *p*-value <0.05.

**Table 4:** Crude and adjusted odds ratios for self-reported falls and balance/walking difficulty in the past 12 months by demographic, health- and cancer-related factors in lung cancer.

Variables	Falls				Balance or Walking Difficulty			
	No (n = 571)	Yes (n = 175)	Crude OR (95% CI)	Adjusted OR (95% CI)	No (n = 377)	Yes (n = 314)	Crude OR (95% CI)	Adjusted OR (95% CI)
Gender								
Men	255	77	1.0	1.0	185	146	1.0	1.0
Women	254	98	1.28 (0.91–1.81)	1.22 (0.74–2.03)	188	165	1.11 (0.82–1.50)	0.69 (0.42–1.14)
Race								
White	411	146	1.0	1.0	306	251	1.0	1.0
Other	106	29	0.77 (0.49–1.21)	0.81 (0.43–1.53)	71	63	1.08 (0.74–1.58)	1.27 (0.70–2.31)
Marital status								
Married	294	95	1.0	1.0	213	174	1.0	1.0
Not married	214	79	1.14 (0.81–1.62)	0.73 (0.45–1.21)	159	135	1.04 (0.77–1.41)	1.05 (0.64–1.72)
Comorbidity	3.2 (1.9)	3.7 (2.0)	**1.14 (1.03–1.26)**^**c**^	0.91 (0.79–1.04)	2.9 (1.8)	3.9 (1.9)	**1.34 (1.22–1.47)**	1.07 (0.93–1.23)
Moderate physical activity								
Not limited	330	71	1.0	1.0	270	129	1.0	1.0
Limited a lot	176	101	**2.67 (1.88–3.80)**^**c**^	1.04 (0.60–1.82)	99	179	**3.78 (2.74–5.23)**^**c**^	1.12 (0.66–1.92)
ADL dependency								
None	282	40	1.0	1.0	258	63	1.0	1.0
1–2 ADL	141	55	**2.75 (1.75–4.33)**^**c**^	1.70 (0.94–3.07)	82	115	**5.74 (3.87–8.53)**^**c**^	**3.39 (2.00–5.72)**^**c**^
3+ ADL	84	72	**6.04 (3.83–9.54)**^**c**^	**4.13 (2.02–8.42)**^**c**^	31	124	**16.38 (10.13–26.48)**^**c**^	**7.14 (3.44–14.80)**^**c**^
Fatigue								
No	305	74	1.0	1.0	254	125	1.0	1.0
Yes	198	96	**2.00 (1.41–2.84**)^**c**^	1.17 (0.68–1.99)	113	181	**3.26 (2.37–4.47)**^**c**^	1.36 (0.81–2.28)
Depression								
No	313	69	1.0	1.0	254	129	1.0	1.0
Yes	157	87	**2.24 (1.74–2.88)**^**c**^	**1.87 (1.12–3.10)**^**a**^	93	149	**3.16 (2.26–4.41)**^**c**^	**1.70 (1.03–2.81)**^**a**^
Urinary incontinence								
No	348	81	1.0	1.0	265	163	1.0	1.0
Yes	163	89	**2.35 (1.65–3.34)**^**c**^	**1.85 (1.16–2.96)**^**a**^	107	144	**2.19 (1.59–3.01)**^**c**^	1.23 (0.77–1.98)
Vision problem								
No	471	146	1.0	1.0	351	266	1.0	1.0
Yes	42	26	**2.00 (1.18–3.37)**^**a**^	1.06 (0.49–2.27)	23	45	**2.58 (1.52–4.37)**^**c**^	1.55 (0.67–3.56)
Hearing problem								
No	426	134	1.0	1.0	320	241	1.0	1.0
Yes	72	38	**1.68 (1.08–2.60)**^**a**^	0.83 (0.45–1.53)	46	63	**1.82 (1.20–2.76)**^**b**^	0.91 (0.50–1.67)
Sensory impairment								
No	391	104	1.0	1.0	313	182	1.0	1.0
Yes	81	50	**2.32 (1.54–3.51)**^**c**^	**1.74 (1.02–2.98)**^**a**^	37	94	**4.37 (2.87–6.66)**^**c**^	**2.84 (1.56–5.17)**^**b**^
Pain interfering with work								
No	256	52	1.0	1.0	222	83	1.0	1.0
Moderate or Severe	255	117	**2.26 (1.56–3.27)**^**c**^	1.34 (0.78–2.31)	148	226	**4.08 (2.95–5.66)**^**c**^	1.57 (0.98–2.52)
Age at diagnosis, y	73.7 (5.6)	74.4 (6.0)	1.02 (0.99–1.05)	**1.05 (1.01–1.09)**^**a**^	73.2 (5.4)	74.8 (6.1)	**1.05 (1.02–1.07)**^**c**^	**1.06 (1.01–1.11)**^**b**^
Survival time since cancer diagnosis, m	31.4 (13.5)	31.7 (13.7)	1.00 (0.99–1.01)	1.01 (0.99–1.02)	31.4 (13.4)	31.5 (13.7)	1.00 (0.99–1.01)	1.01 (0.99–1.03)
Cancer stage								
In situ	0^d^	0^d^			0^d^	0^d^		
Localized	261	84	1.0	1.0	197	147	1.0	1.0
Regional	209	68	1.01 (0.70–1.46)	0.65 (0.38–1.11)	159	119	1.00 (0.73–1.38)	0.76 (0.45–1.28)
Distant	98	32	1.02 (0.64–1.62)	1.24 (0.61–2.53)	65	64	1.32 (0.88–1.98)	1.19 (0.58–2.46)
Surgery								
No	203	80	1.0	1.0	137	146	1.0	1.0
Yes	313	95	0.77 (0.55–1.09)	1.34 (0.70–2.56)	239	168	**0.66 (0.49–0.90)**^**b**^	0.95 (0.50–1.79)
Radiation								
No	363	121	1.0	1.0	268	215	1.0	1.0
Yes	149	53	1.07 (0.73–1.55)	1.52 (0.81–2.86)	106	96	1.13 (0.81–1.57)	0.87 (0.47–1.64)

Abbreviation: ADL, activities of daily living.

Values show are number, mean (SD), or ORs (95%CI).

Statistically significant ORs are marked in bold text with numerical superscripts indicating the level of significance.

^a^ *p*-value <0.05,

^b^ *p*-value <0.05, and

^c^ *p*-value <0.05.

^d^ There were no survivors with the stage “in situ”.

**Table 5:** Crude and adjusted odds ratios for self-reported falls and balance/walking difficulty in the past 12 months by demographic, health- and cancer-related factors in prostate cancer.

Variables	Falls				Balance or Walking Difficulty			
	No (n = 3094)	Yes (n = 759)	Crude OR (95% CI)	Adjusted OR (95% CI)	No (n = 3085)	Yes (n = 1148)	Crude OR (95% CI)	Adjusted OR (95% CI)
Race								
White	2305	604	1.0	1.0	2102	796	1.0	1.0
Other	707	135	**0.73 (0.59–0.89)**^**b**^	**0.67 (0.51-.0.87)**^**b**^	613	229	0.99 (0.83–1.17)	0.86 (0.67–1.11)
Marital status								
Married	2677	489	1.0	10	2064	695	1.0	1.0
Not married	375	256	**1.54 (1.30–1.83)**^**c**^	**1.29 (1.04–1.60)**^**a**^	676	341	**1.50 (1.28–1.75)**^**c**^	1.09 (0.87–1.36)
Comorbidity	2.3 (1.7)	3.3 (2.0)	**1.33 (1.27–1.40)**^**c**^	**1.11 (1.05–1.18)**^**b**^	2.1 (1.6)	3.5 (1.9)	**1.53 (1.46–1.60)**^**c**^	**1.14 (1.07–1.21)**^**c**^
Moderate physical activity								
Not limited	1463	383	1.0	1.0	2515	646	1.0	1.0
Limited a lot	352	245	**3.74 (3.10–4.50)**^**c**^	**1.41 (1.07–1.86)**^**a**^	235	389	**6.44 (5.36–7.74)**^**c**^	**1.46 (1.10–1.94)**^**b**^
ADL dependency								
None	2255	286	1.0	1.0	2242	297	1.0	1.0
1–2 ADL	526	204	**3.06 (2.50–3.75)**^**c**^	**1.95 (1.49–2.54)**^**c**^	361	364	**7.61 (6.30–9.20)**^**c**^	**3.32 (2.60–4.24)**^**c**^
3+ ADL	230	243	**8.33 (6.70–10.36)**^**c**^	**3.73 (2.69–5.19)**^**c**^	115	355	**23.30 (18.28–29.70)**^**c**^	**6.12 (4.40–8.51)**^**c**^
Fatigue								
No	2533	465	1.0	1.0	2411	583	1.0	1.0
Yes	503	281	**3.04 (2.44–3.63)**^**c**^	1.09 (0.83–1.42)	323	453	**5.80 (4.90–6.87)**^**c**^	**1.48 (1.14–1.92)**^**b**^
Depression								
No	2305	426	1.0	1.0	2158	569	1.0	1.0
Yes	554	264	**2.51 (1.75–3.59)**^**c**^	**1.28 (1.01–1.62)**^**a**^	424	390	**3.49 (2.96–4.12)**^**c**^	1.21 (0.95–1.54)
Urinary incontinence								
No	1822	304	1.0	1.0	1699	425	1.0	1.0
Yes	1228	442	**2.16 (1.83–2.54)**^**c**^	**1.54 (1.25–1.88)**^**c**^	1045	615	**2.35 (2.03–2.72)**^**c**^	**1.64 (1.34–2.01)**^**c**^
Vision problem								
No	2895	668	1.0	1.0	2644	908	1.0	1.0
Yes	174	81	**2.02 (1.53–2.66)**^**c**^	0.91 (0.61–1.34)	117	136	**3.39 (2.61–4.38)**^**c**^	**1.74 (1.17–2.59)**^**b**^
Hearing problem								
No	2524	563	1.0	1.0	2327	750	1.0	1.0
Yes	478	177	**1.66 (1.37–2.02)**^**c**^	1.03 (0.79–1.33)	375	279	**2.31 (31.94–2.75)**^**c**^	1.07 (0.82–1.39)
Sensory impairment								
No	2566	532	1.0	1.0	2401	690	1.0	1.0
Yes	295	156	**2.55 (2.06–3.16)**^**c**^	1.26 (0.95–1.66)	186	263	**4.92 (4.00–6.05)**^**c**^	**1.84 (1.39–2.45)**^**c**^
Pain interfering with work								
No	2089	310	1.0	1.0	2055	341	1.0	1.0
Moderate or Severe	970	432	**3.00 (2.55–3.54)**^**c**^	1.07 (0.84–1.38)	698	697	**6.02 (5.15–7.03)**^**c**^	**1.80 (1.43–2.28)**^**c**^
Age at diagnosis, y	72.1 (5.2)	74.1 (6.3)	**1.06 (1.05–1.08)**^**c**^	**1.04 (1.02–1.06)**^**c**^	71.8 (5.1)	74.1 (6.2)	**1.08 (1.06–1.09)**^**c**^	**1.05 (1.04–1.07)**^**c**^
Survival time since cancer diagnosis, m	35.8 (13.8)	36.1 (13.7)	1.00 (1.00–1.01)	1.00 (0.99–1.01)	35.8 (13.8)	36.0 (13.8)	**2.35 (1.98–2.78)**^**c**^	1.00 (0.94–1.01)
Cancer stage								
In situ	0^d^	0^d^			0^d^	0^d^		
Localized	3048	736	1.0	1.0	2750	1023	1.0	1.0
Regional	326	53	0.81 (0.55–1.19)	0.81 (0.55–1.19)	290	89	0.83 (0.64–1.06)	1.15 (0.79–1.67)
Distant	55	26	1.36 (0.74–2.48)	1.36 (0.74–2.48)	45	36	**2.15 (1.38–3.35)**^**b**^	0.85 (.0.44–1.63)
Surgery	2164	570						
No	900	182	1.0	1.0	1917	806	1.0	1.0
Yes			**0.77 (0.64–0.92)**^**b**^	1.11 (0.84–1.47)	846	236	**0.66 (0.56–0.78)**^**c**^	0.89 (0.67–1.18)
Radiation	1637	423						
No	1402	325	1.0	1.0	1452	599	1.0	1.0
Yes	2164	570	0.90 (0.76–1.05)	1.19 (0.93–1.50)	1286	438	**0.83 (0.72–0.95)**^**b**^	0.93 (0.73–1.18)

Abbreviation: ADL, activities of daily living.

Values show are number, mean (SD), or ORs (95%CI).

Statistically significant ORs are marked in bold text with numerical superscripts indicating the level of significance.

^a^ *p*-value <0.05,

^b^ *p*-value <0.05, and

^c^ *p*-value <0.05.

^d^ There were no survivors with the stage “in situ”.

Tables 2–5 also present the results from univariable analyses of factors linked to balance or walking difficulty. In colorectal cancer, women were more likely to report balance/walking difficulty than men. Being unmarried was associated with balance/walking difficulty in breast, colorectal, and prostate cancer. All health-related factors and advancing age at cancer diagnosis were associated with balance/walking difficulty in all cancer types. Regional or distance cancer stage in breast cancer and distance cancer stage in prostate cancer were associated with difficulty in balance/walking. In both breast and prostate cancer, treatment with surgery and radiation was associated with reduced odds of balance/walking difficulty. Surgery also was associated with reduced balance/walking difficulty in lung cancer.

### Multivariable analyses

Multivariable regression revealed that ADL dependence and advancing age at cancer diagnosis were independent factors for falls and balance/walking difficulty in all cancer types (Tables 2–5). Comorbidity was another significant independent factor linked to falls and balance/walking difficulty in breast, colorectal, and prostate cancer. Additionally, in all cancer types, depression was independently associated with falls, whereas sensory impairment was independently associated with balance/walking difficulty. Urinary incontinence was significantly associated with falls in breast, lung, and prostate cancer. In breast cancer, localized or regional cancer stage was an independent factor for increased falls and regional cancer stage was linked to balance/walking difficulty. Radiation was associated with reduced falls in breast cancer.

## Discussion

The impact of falls and balance/walking difficulty on health outcomes may be under-recognized in older cancer survivors. Only 10% of survivors with a recent fall had their falls documented by oncology clinicians [35]. Balance/walking difficulty is rarely recorded in outpatient oncology settings [12]. Current findings bring important insight about factors related to falls and balance/walking difficulty within each of four major cancers in older survivors based on population-based data. This study suggests significant factors that may be integrated into clinical screening algorithms to identify older breast, colorectal, lung, and prostate cancer survivors at risk of falling or developing balance and walking difficulty. In order to optimize function and reduce long-term disability, fall prevention in cancer survivors has been recommended as the top priorities by key stakeholders, including National Cancer Institute, National Center for Medical Rehabilitation Research, and American Congress of Rehabilitation Medicine [36,37]. Screening is an essential first step in preventing falls and improving balance or walking ability after cancer diagnosis.

We found that among older survivors who were one to five years after the diagnosis of breast, colorectal, lung, or prostate cancer, 20%-26% reported falls and 28%-45% reported having balance/walking difficulty in the past 12 months. Analyses of Medicare/Medicaid beneficiaries showed that 26%-33% of older cancer survivors had falls [10,11]. Methods for measuring falls in previous studies were unclear [10,11]. MHOS collects information of falls by asking about the occurrence of falls in the past 12 months [29]. Recall bias and underreporting of falls [38] likely contributed to lower fall rates in MOHS data. Previous research reported that 46% of Medicare beneficiaries had mild-severe limitation in walking as determined by four questions about difficulty, assistive devices, and need for assistance during walking [39]. This study determined balance/walking difficulty based on one MHOS question, which may not reflect the full spectrum of balance skills and walking ability.

In this study, univariable analyses showed that a majority of factors associated with falls and balance/walking difficulty are similar across four cancer types. However, after multivariable analyses, independent factors of falls and balance/walking difficulty that are unique to each cancer type emerged. Additionally, within each cancer types, not all independent factors of falls were linked to balance/walking difficulty and vice versus. Cancer sequelae are specific to each cancer type [40] and therefore, likely have different impacts on body functions that are important in preventing falls and controlling balance and walking. Falls and balance/walking difficulty may have some common determinants, but each has a different construct. The control of balance skills and walking requires postural alignment, muscle strength, perceived stability limit, sensory integration, anticipatory and reactive postural reactions, and cognition [41]. While impairment in balance and walking is a major risk factor of fall, the causes of falls is multifactorial and involve other factors, such as prior falls, age, race, gender, medications, use of assistive devices, comorbidities, depression, and environmental hazards [3,17]. Research of the general geriatric population has demonstrated that the relationship between falls and walking ability as measured by gait speed is not linear [42]. Older adults with higher and slower gait speed were more likely to fall than those with normal gait speed [42]. Taken together, risk factor profiles of falls and balance/walking difficulty in older survivors likely differ within each cancer type. Falls and balance/walking difficulty may need to be examined as major health outcomes separately.

White race was independently associated with falls in breast and prostate cancer. Race was not an independent factor of balance/walking difficulty in any cancer type. Previous research of older cancer survivors did not find fall rates to differ between white race vs. others [19,43]. In cancer survivors treated with chemotherapy, white race was associated with increased risk of injurious falls [44]. A U.S. population-based study revealed that the percentages of older adults with falls in the past 3 months were 27.8% in American Indian, 17.4% in Hispanic, 13% in black/Asian/pacific islanders, and 15.8% in white [45]. This low fall rate in whites in the general geriatric population was in contrast to current findings of an increased odd of falls in white survivors. In early stage breast cancer, racial disparities in treatments have been reported, with black less likely to received standard care than white [46]. Recent population-based research with a larger sample size reported that in late stage breast cancer, racial difference in cancer treatment was not observed [47]. Additionally, non-significant borderline associations indicated that white women were more likely to receive chemotherapy and had worse mortality rate in comparison with black women [47]. Whether the interplay between race and cancer treatment affects the outcomes of falls and balance/walking difficulty was not examined in this study, and warrant further research.

In prostate cancer only, married prostate cancer survivors were less likely to report falls than those who were unmarried. Marital status, however, was not linked to balance/walking difficulty. In older men with prostate cancer, marriage was found to protect against inflammation [48] and promote behaviors in seeking healthcare [49]. In comparison with those who were not married, married prostate cancer survivors might have received more care and support to reduce fall risk, such as using assistive device, checking footwear, and modifying home environment for safety. Supportive care to reduce fall risk may be pertinent in older, unmarried prostate cancer survivors.

In older adults, female gender is a significant predictor of falls [3], while risk factors for falls differ between genders [50]. Among older cancer survivors treated with neurotoxic chemotherapy, women were more likely to fall than men [44]. We did not find gender to be independently associated with falls or balance/walking difficulty in colorectal or lung cancer.

Similar to prior studies [10,19,22,44,51,52], we found significant associations of health-related factors with falls and balance/walking difficulty. ADL dependence was the only independently factor linked to both falls and balance/walking difficulty in breast, colorectal, lung, and prostate cancer. In all four cancer types, depression was an independent factor of falls whereas sensory impairment was an independent factor of balance/walking difficulty. Other health-related factors linked to falls or balance/walking difficulty differed across cancer types. Current findings underscore the need to manage impairments, functional deficits, and burden from comorbidity that may be associated with falling in older cancer survivors. Research suggested integrating comprehensive geriatric assessment (CGA) into oncology practice to guide cancer treatments and survivorship care [53–55]. CGA identifies comorbidity and functional impairments that may increase fall risk and warrant referrals for rehabilitation [19,56].

Except for lung cancer, comorbidity was an independent factor of both falls and balance/walking difficulty in other cancer types. Lung cancer survivors are more likely to be diagnosed at advanced stages and receive aggressive cancer treatments resulting in a multitude of side effects [40]. It is possible that disease burden and symptoms from lung cancer itself has a larger impact on overall body function than other comorbidity. Findings from current and prior studies of cancer survivors showed that with each additional comorbidity, the odds of reporting falls increased by 10%-20% [19]. In the general geriatric population, comorbidity is also an important risk factor of falls [3] and walking difficulty [39]. Impairment from some chronic conditions, such as arthritis and stroke, increases fall risk [19,57]. This study only examined the total number rather than the type of chronic conditions with relaiton to fall or balance/walking difficulty. Previous research of the SEER-MHOS data reported that after a cancer diagnosis, older cancer survivors developed different types of chronic conditions depending on the cancer type [58]. For example, in prostate cancer, musculoskeletal, major depression, gastrointestinal and pulmonary coditions were the most prevalent conditions. In breast cancer, the prevalence of diabetes and high blood pressure were the highest [58]. Future research is necessary to elucidate the impact of newly developed chronic conditions after a cancer diagnosis on falls and balance/walking difficulty within each cancer type.

We found that ADL dependence was independently linked to falls and balance/walking difficulty in all cancer types. Compared to those without ADL dependence, survivors with ADL dependence had two- to four-fold increase in fall risk. Additionally, ADL dependence increased the risk for balance/walking difficulty by three to thirteen times. In older adults, both improvement and deterioration in ADL over 6 years had been linked to falls [59]. In older cancer survivors of mixed cancer types, balance/walking difficulty was significantly associated with distress in managing household activities [16]. Individuals with improved ADL may be exposed to higher fall risk due to better mobility and function [59]. Incorporate ADL training into fall-prevention and balance/walking intervention programs may need to be considered in older cancer survivors.

Depression was independently associated with falls in all cancer types, and with balance/walking difficulty in breast, colorectal, and lung cancer. Depression is associated with decreased physical activity, sleep disorder, cognitive impairment, and use of antidepressant, which collectively increase fall risk in older adults [60]. Individuals with depression had impaired balance response, which may be related to deficits in integration of visual and proprioceptive inputs to control posture [61,62]. Previous research of older survivors of mixed cancer types reported that depression increased the odds of falling [63]. However, in older cancer survivors who were receiving chemotherapy, depression was not associated with falls [56]. Because the SEER-MHOS linkage does not include chemotherapy data, this study could not determine whether the impact of depression varies by cancer treatment. No other study has examined the relationship between depression and balance/walking difficulty in older cancer survivors. Urinary incontinence was independently associated with falls in breast, lung, and prostate cancer. In breast and prostate cancer, urinary incontinence was also an independent factor of balance/walking difficulty. Previous research identified urinary incontinence as a risk factor for falls in older survivors with mixed cancer diagnoses [51] or advanced cancer [23]. Urinary incontinence is a complication of prostate cancer treatment [64]. Prevalence of urinary incontinence is higher in breast and prostate cancer survivors compared to non-cancer controls [65] Urinary incontinence is recognized as a geriatric syndrome that often co-exists with other chronic conditions [66].

In all cancer types, sensory impairment was an independent factors of balance/walking difficulty. In colorectal and lung cancer only, sensory impaired was independently linked to falls. We defined sensory impairment as numbness, tingling, burning, loss of decreased ability to feel in the feet in the previous 4 months. Sensory impairment is a common complication of neurotoxic chemotherapy and contributes to balance and walking problems [67]. In breast cancer survivors, poor performance in balance and walking was attributed to impaired ability to integrate sensory information from visual, vestibular, and somatosensory systems [24]. SEER-MHOS does not provide the information about chemotherapy. As such, whether sensory impairment observed in this study was related to chemotherapy or other conditions, such as peripheral neuropathy from diabetes [68,69], cannot be acertained.

The extent of pain interfering with work was not associated with falls in all cancer types. A study of older cancer survivors showed that pain measured by Visual Analog Scale was linked to falls [52]. Cancer-related pain is an important factor associated with falls in survivors with advanced cancer [23]. We determined pain based on one MHOS question asking how often in the past 4 weeks, pain interfered with normal work. The question did not identify the location or nature of the pain. A comprehensive questionnaire specific to cancer, such as Edmonton Classification System for Cancer Pain, may elucidate the relationship between pain and falls in cancer survivors [23]. This study found that pain was an independent factor of balance/walking difficulty in prostate cancer only. In localized prostate cancer survivors, 69.6% reported pain in the past 3 months [70]. Additionally, 72.5% of pain was located at back, lower abdomen, lower extremity, or hip.^69^ Older prostate cancer survivors may encounter more difficulty in balance and walking because pain is prevalent and primarily involves the lower body in this population.

In this study, age at cancer diagnosis was the only cancer-related independent factor associated with falls and balance/walking difficulty in all cancer types. Age is a risk factor for falling in older adults [3]. We found that the odds for reporting falls and balance/walking difficulty increased by 3%-11% and by 2%-6%, respectively, with each additional year of age at cancer diagnosis. In survivors with advanced cancer or chemotherapy induced peripheral neuropathy, age did not predict falls [23,67]. Age, specifically chronological age as measured in this study, is unlikely the key driver of falls and balance/walking difficulty in older cancer survivors. Research of SEER-MHOS linkage reported that older cancer survivors had greater declines in physical function over time than age-matched controls [71]. Cancer treatment has long-lasting impact on non-tumor cells. Vulnerabilities of genome instability, epigenetic changes, and cellular senescence likely interact with age, leading to accelerated aging observed in cancer survivors [72].

In breast cancer only, localized or regional cancer stage was independently associated with increased odds of falls and balance/walking difficulty. Previous research reported no associations between falls with cancer stage, cancer type, or time post-cancer diagnosis in older survivors of mixed cancer types [10], and in postmenopausal breast cancer survivors [73]. Treatments for advanced breast cancer, particularly neurotoxic chemotherapy, cause a wide variety of side effects affecting body systems. Medications prescribed to manage symptoms in advanced cancer, such as anticonvulsants, antidepressants, and antipsychotics [23,74], are known to increase fall risk and affect physical function in older adults [75]. Cancer symptoms and treatment complications specific to each cancer types likely influence the associations of cancer stage with falls and balance/walking.

In breast cancer only, radiation was independently associated with reduced odds of falls, but not balance/walking difficulty. Radiotherapy is the local treatment of choice in breast cancer. Radiotherapy improved long-term outcomes and survival by reducing breast cancer recurrence and mortality [76,77]. This study confirmed additional benefit of radiation by reducing fall risk in breast cancer. Reasons for omitting radiotherapy, such as cultural beliefs, limited access to healthcare, previous breast irradiation, inability to lie flat or abduct the arm, or having collagen vascular disease [78,79] were not available from SEER-MHOS linkage [29].

This study has limitations. First, because of cross-sectional design, associations between factors with falls and balance/walking difficulty cannot be construed as causal relationships. Second, SEER-MHOS does not include information about chemotherapy or hormonal therapy [29]. Third, the time when a condition or impairment developed cannot be identified from SEER-MHOS data. Fourth, primary outcomes were obtained by self-report and subject to recall bias [38]. Lastly, MHOS randomly surveyed Medicare Advantage beneficiaries, which have more risk factors and lower function than other Medicare beneficiaries [80].

## Conclusion

Each of the four cancer types being examined in this study has unique patterns of demographics, health- and cancer-related factors that are independently linked to falls and balance/walking difficulty. In breast, colorectal, lung and prostate cancer, ADL dependence and age at cancer diagnosis were common independent factors linked to both falls and balance/walking difficulty. In all four cancer types, depression was an independent factor of falls whereas sensory impairment in feet was an independent factor of balance/walking difficulty. These findings have important implications in screening for older cancer survivors at risk of falls and balance/walking difficulty.
